# Flexible Graphene Electrodes for Prolonged Dynamic ECG Monitoring

**DOI:** 10.3390/s16111833

**Published:** 2016-11-01

**Authors:** Cunguang Lou, Ruikai Li, Zhaopeng Li, Tie Liang, Zihui Wei, Mingtao Run, Xiaobing Yan, Xiuling Liu

**Affiliations:** 1 Department of Biomedical Engineering, College of Electronic Information Engineering & Key Laboratory of Digital Medical Engineering of Hebei Province, Hebei University, Baoding 071002, China; loucunguang@163.com (C.L.); 15031286182@163.com (R.L.); 15712590824@163.com (Z.L.); lanswer@163.com (T.L.); 2 College of Quality and Technical Supervision, Hebei University, Baoding 071002, China; zihui-wei@163.com; 3 College of Chemistry & Environment Science, Hebei University, Baoding 071002, China; lhbx@hbu.edu.cn; 4 Department of Electronic Science and Technology, College of Electronic Information Engineering & Key Laboratory of Digital Medical Engineering of Hebei province, Hebei University, Baoding 071002, China; xiaobing_yan@126.com

**Keywords:** graphene, ECG, flexible electrode, home health

## Abstract

This paper describes the development of a graphene-based dry flexible electrocardiography (ECG) electrode and a portable wireless ECG measurement system. First, graphene films on polyethylene terephthalate (PET) substrates and graphene paper were used to construct the ECG electrode. Then, a graphene textile was synthesized for the fabrication of a wearable ECG monitoring system. The structure and the electrical properties of the graphene electrodes were evaluated using Raman spectroscopy, scanning electron microscopy (SEM), and alternating current impedance spectroscopy. ECG signals were then collected from healthy subjects using the developed graphene electrode and portable measurement system. The results show that the graphene electrode was able to acquire the typical characteristics and features of human ECG signals with a high signal-to-noise (SNR) ratio in different states of motion. A week-long continuous wearability test showed no degradation in the ECG signal quality over time. The graphene-based flexible electrode demonstrates comfortability, good biocompatibility, and high electrophysiological detection sensitivity. The graphene electrode also combines the potential for use in long-term wearable dynamic cardiac activity monitoring systems with convenience and comfort for use in home health care of elderly and high-risk adults.

## 1. Introduction

Cardiovascular disease, which includes both coronary heart disease and stroke, is one of the most prevalent causes of death worldwide. In addition to these fatal cases, at least 20 million people experience nonfatal heart attacks and strokes every year; many of these people subsequently require prolonged and costly medical care [1,2]. Electrocardiography (ECG), which records the electrical activity of the patient’s heart over time to obtain important diagnostic information, has served as a useful diagnostic tool in modern clinical medicine. Routine and repetitive ECG measurements are often necessary for cardiovascular patients. However, the inconvenience to the patient, the high cost of hospital-centered care, and the demand for long-term recording methods have combined to drive significant interest in homecare development [3]. In particular, as the elderly population gradually increases in more and more countries, home health care management for these elderly people has attracted increasing attention. With the continuing advances in device miniaturization and wireless technologies, wearable on-body ECG devices for long-term use that have been devised to capture and monitor the electrical activity of the heart may help to establish diagnoses in patients, and considerable progress has been made in this field [4,5,6,7]. However, despite advancements in wireless technology and electronics miniaturization, the development of daily ECG monitoring equipment is still limited by the inconvenience of and patient discomfort caused by wet adhesive electrodes [8], which has led to new requirements for high-performance ECG electrodes with biological compatibility. The standard commercial Ag/AgCl electrode used for ECG signal detection relies on a conductive gel to maintain good electrical contact with the skin, but this gel often dries out over time, causing impedance variations and a dramatic reduction in signal quality. In addition, the gel can irritate the patient’s skin, and thus cause excessive discomfort. Sweat provides another source of signal degradation for wet electrodes [9]. These problems make the conventional Ag/AgCl wet electrode unsuitable for both routine and repetitive ECG measurements in home health care systems.

Following the development of flexible and stretchable electronics, alternative electrodes that do not require electrolytic gel (referred to as active dry electrodes) have been reported by many groups [9,10,11,12,13,14,15,16]. Baek proposed flexible polymeric electrodes [9], and Lee developed a metallic material layer, carbon nanotube (CNT) and polydimethylsiloxane (PDMS) composite-based dry ECG electrode [10]. Ruffini proposed a dry electrode based on multiwall CNT (MWCNTs) arrays to penetrate the patient’s outer skin cell layers and reduce the measurement noise [12]. Park proposed capacitive non-contact sensors based on printed-circuit board technology [15]. Graphene, the recently discovered two-dimensional (2D) carbon allotrope, has received considerable interest in many scientific fields because of its fascinating properties, which include excellent biocompatibility, superior electrical conductivity, high thermal conductivity, and extraordinary elasticity and stiffness [17,18,19,20,21]. Graphene is a highly promising material for use in the development of flexible electronics and wearable ECG sensors. Recently, Yapici united graphene with ordinary textiles to develop graphene-clad, conductive textile electrodes that enabled the acquisition of high quality ECG signals [22]. Kim developed a single stretchable and conductive dry adhesive electrode; its excellent cycling properties suggest a suitable strategy for repeatable measurements of biosignals under daily activity conditions [23]. Celik presented a graphene-based electrode by coating graphene on top of a metallic layer of an Ag/AgCl electrode for acquisition of ECG [24]; better performance was obtained than with conventional ECG electrodes. While the breathability of PDMS and the comfort of the metallic material layer should be improved for long-term monitoring, a low-complexity-preparation technology with improved degree of adhesion would be far more valuable and worth the effort to develop.

In this study, flexible graphene textile was synthesised by the reduction of graphene oxide with the help of vacuum filtration to improve the amount and degree of adsorption, and then the graphene textile was used to construct electrodes for long-term wearable ECG monitoring applications. The structural properties of graphene were first characterized by Raman spectroscopy and scanning electron microscopy (SEM), and then the electrical properties of the designed electrode were evaluated by diverse testing based on alternating current (AC) impedance spectroscopy and connect impedance with skin, according to frequency changes. Subsequently, we measured ECG signals in different states of human body motion and studied the feasibility of long-term monitoring through continuous measurements. The experimental result and statistical comparative study demonstrates the potential usefulness of the proposed graphene electrodes for routine electrophysiological activity monitoring of the heart and other vital organs.

## 2. Materials and Methods

### 2.1. Wireless ECG Monitoring System

**Figure 1:**
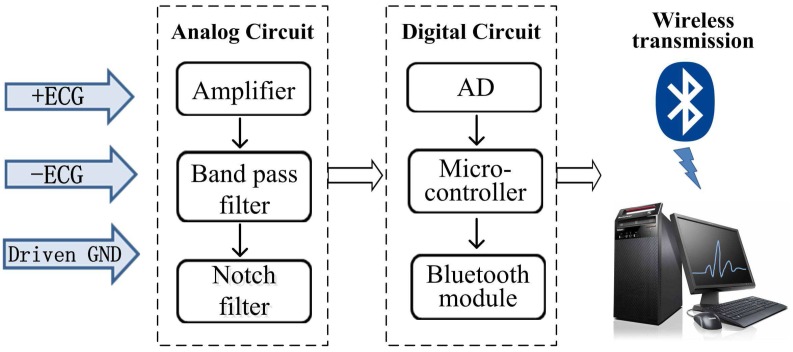
Block diagram of electrocardiograph (ECG) signal processing and data acquisition system. AD: analog-to-digital converter, GND: ground.

**Figure 2:**
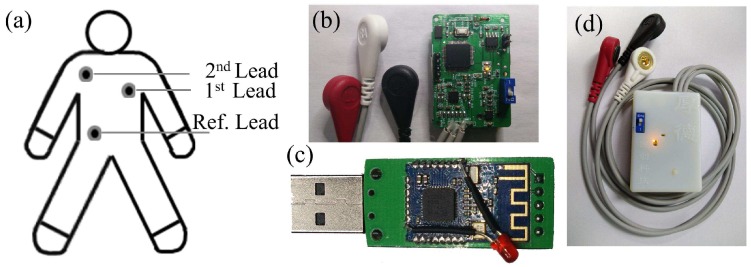
(**a**) Schematic illustration of the three-lead electrodes; (**b**) photograph of the data collection and Bluetooth communication module; (**c**) data reception module; (**d**) photograph of the assembled ECG holter.

Figure 1 shows the designed ECG monitoring system, which consists of electrodes, data preprocessing, analog-to-digital (AD) conversion, wireless communication modules, and a personal computer (PC)-based data-processing platform. The electrical activity of the heart was first converted into electrical signals by the electrodes, and these signals were then amplified by the single-lead AD8232 analog front with a gain of 62 dB. The processed signals were converted into digital signals by the MSP430F149 with a sampling frequency of 200 Hz, and then were transmitted to a Java platform through a Bluetooth communication module (CC2541); the detected ECG signals were then displayed and analyzed on the computer in real time. Additionally, the detected ECG signal data can be stored automatically using an onboard Secure Digital (SD) card for further analysis and diagnosis. A band-pass filter with a transmission frequency range from 0.05 to 100 Hz and a 50 Hz notch filter were designed as part of the program to reduce noise fluctuations and power frequency interference. Figure 2a shows that the three-lead electrodes were in contact with the heart to record the patient’s ECG signals. Figure 2b,c show the circuit boards of the designed data collection, Bluetooth communication, and the data reception module, respectively. Figure 2d shows a photograph of the assembled ECG holter.

### 2.2. Construction of the Graphene Electrode

Numerous methods can be used to fabricate graphene, including chemical vapor deposition (CVD) [24], organic methods, and reduction of graphene oxide [25,26,27]. In this paper, graphene paper with a thickness of 60 μm (XFNano Materials Tech., Nanjing, China) and graphene films with thicknesses of several nm (Six Carbon Tech., Shenzhen, China; Vigon Materials Tech., Hefei, China) were first used for construction of the graphene electrode. Then, we synthesized a flexible graphene textile electrode by the reduction of graphene oxide (GO) (XFNano Materials Tech.) using hydrazine hydrate (AlfaAesar) as a reducing agent. The GO suspension was deposited on gaps in a polyester fiber (200 D) by vacuum filtration, which provides obvious enhancement in terms of the adsorption amount and adsorptive degree when compared with previously reported thermal evaporation methods, and leads to improved conductivity.

**Figure 3:**
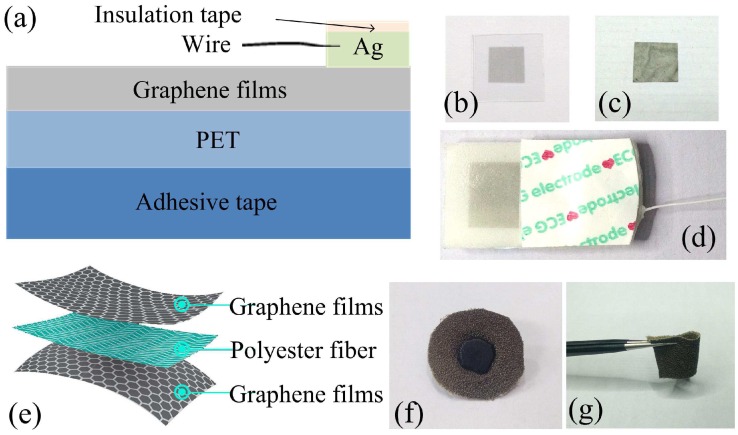
(**a**) Schematic diagram of flexible dry graphene electrode; (**b**,**c**) photographs of graphene-polyethylene terephthalate (PET) and graphene paper; (**d**) assembled electrode; (**e**) schematic diagram of graphene textile; (**f**,**g**) photographs of graphene textile electrode.

Figure 3a shows the structure of the graphene electrode on the polyethylene terephthalate (PET) substrate. The graphene films were first grown on copper foils by CVD and were then transferred to a flexible PET substrate with a thickness of 280 μm. Photographs of the graphene–PET structure and the graphene paper are shown in Figure 3b,c, respectively; the dimensions are 10 mm × 10 mm. We fabricated the ECG electrode by connecting a silver wire to the graphene using conductive silver pulp, and encapsulated the resulting conductive node with insulating glue to prevent direct contact with the skin, as shown in Figure 3d. During the measurements, the assembled electrode was fixed to the patient’s chest using bandages. Figure 3e shows the structure of the designed graphene textile, where the graphene layers were adsorbed on the top and bottom surfaces of the flexible polyester fiber. The assembled graphene textile electrode is shown in Figure 3f; a plastic fastener passes through the middle of the graphene textile and is connected to the underlying surface using a metal snap for convenient connection of the electrode to a commercial ECG cable. Figure 3f,g show that the textile electrode is highly flexible, and is suitable for fixing to the ribcage with bandages or using a waistcoat when performing dynamic ECG measurements.

### 2.3. Characterization of Structural and Electrical Properties

Raman spectroscopy and SEM are widely used characterization methods for examination of the microstructures of materials. Raman spectroscopy provides information about the characteristic vibrational states, while SEM gives surface profile information. In this paper, the Raman spectra of graphene electrodes were measured using a Horiba Jobin Yvon confocal LabRAM HR800 spectrometer with an excitation wavelength of 532 nm, and the SEM was performed using a Hitachi TM3030 electron microscope with an accelerating voltage range of 3–15 kV. The electrical properties of the ECG electrodes were characterized via electrochemical impedance spectroscopy (EIS) measurements that were performed using a CS350 electrochemical workstation (Corrtest Instrument, Wuhan, China), which worked with a sweeping frequency range from 50 mHz to 10 Hz and an AC perturbation of 10 mV. Skin–electrode contact impedance measurement has always been of interest due to the desire to prove the reliability of the collected biopotential [24]. Here, the impedances of standard Ag/AgCl electrode and graphene textile electrode were measured on a person’s forearm by a Precision RLC bridge (QuadTech Type 1693, Canal Winchester, OH, USA). The measurement voltage was 1 V, and the frequency range was from 10 Hz to 1000 Hz. The electrodes were placed adjacent to each other with a distance of 10 cm. All the impedance measurements were replicated 10 times, and the average result value taken.

### 2.4. Motion Artifacts Evaluation and Long-Term ECG Measurement

For performance evaluation of the graphene electrode, ECG signals were measured in different human body motion states in 24-year-old male volunteers. Two electrodes were fixed on the left and right sides of the ribcage of each test subject, and the reference ground was located in their lower right abdomen. To ensure similar test conditions for comparison, these electrodes were positioned in the same locations for every measurement. The resting-state ECG signals were measured first, and then the motion artifacts that result from walking and swinging of the subject’s arms were investigated. To study the effects of moisture on the quality of the ECG signals, measurements were also taken after exercise. Finally, the ECG signals were measured using the graphene textile electrodes over a period of one week to evaluate their long-term monitoring capabilities.

## 3. Results

**Figure 4:**
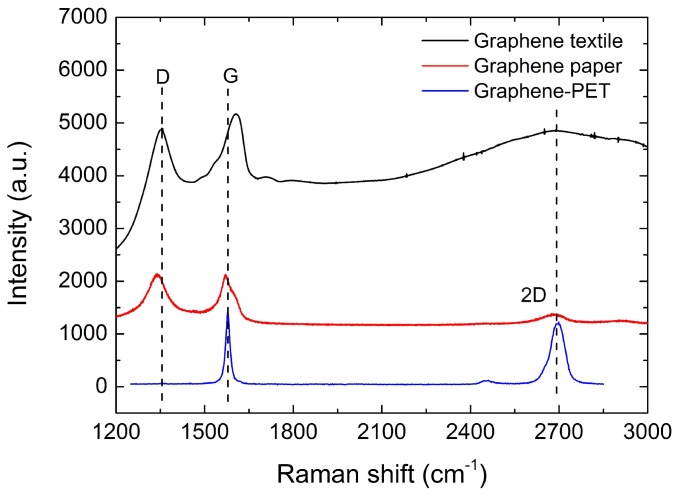
Raman spectra of graphene textile (**black line**), graphene paper (**red line**), and graphene–PET structure (**blue line**).

The Raman spectra of graphene for various thicknesses are shown in Figure 4. As shown in this figure, the Raman resonances of the CVD-produced graphene–PET films are commonly observed at the G-peak (1587 cm${}^{-1}$) and the 2D-peak (2682 cm${}^{-1}$). For the narrow, symmetrical 2D peaks, the value of full width at half maximum is approximately 40 cm${}^{-1}$. The 2D-to-G-peak amplitude ratio (2D/G) acts as a sensitive probe value for monitoring of the effects of electron-donor and electron-acceptor molecules on the electronic properties of graphene; the ratio has often been used to estimate the graphene thickness [28,29], and the 2D/G ratio of this graphene–PET structure reached as high as 0.83. The strong peaks of the G and 2D bands and the strongly-suppressed defect-related D band are well matched with those of typical single-layer graphene, which indicate the high quality of the few-layer graphene films in the PET.

In the graphene textile and the graphene paper, the Raman spectra show two main peaks, the G- and D-peaks, which lie at approximately 1580 cm${}^{-1}$ and 1350 cm${}^{-1}$, respectively. The D band is only activated in the presence of defects, and the defects in the graphene can be assessed through measurement of the intensity ratio of the defect-induced D band to the graphenic G band [29]. Compared with the graphene–PET films, the D band intensity is significantly increased, and the values of I${}_{D}$/I${}_{G}$ for the two materials are estimated to be 0.95 and 1.03, respectively. However, the figure shows broadening of the 2D peaks and a reduction in the relative intensity of the 2D peak, and the 2D/G ratios for the graphene textile and the graphene paper are 0.68 and 0.21, respectively; this indicates the multilayer characteristics and the structural defects that are caused by the reduction processes.

**Figure 5:**
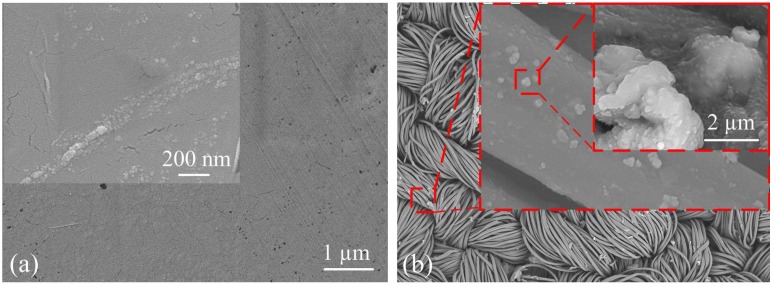
Scanning electron microscope images of (**a**) graphene–PET structure and (**b**) graphene textile.

The morphological studies of the graphene samples performed using SEM are shown in Figure 5. Figure 5a presents SEM micrographs of the graphene films on PET, where the relatively homogenous dispersion of the layer-structured film was observed. Figure 5b shows polyester fibers clad with the reduced graphene oxide, in which some discontinuous microstructures were observed, and the stack-like morphology can be clearly seen in the inset figure, which shows a close-up image of a single polyester fiber surface. Small aggregates were also formed because of the extremely high specific area of GO and the strong particle–matrix interactions that occur.

**Figure 6:**
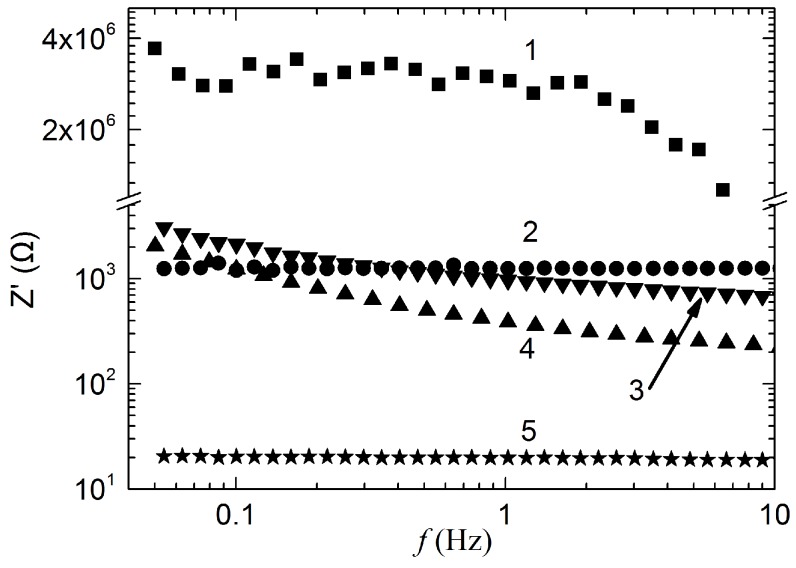
Frequency-dependent impedances of the different ECG electrodes, 1: graphene textile; 2: graphene–PET; 3: Ag/AgCl dry; 4: Ag/AgCl wet; 5: graphene paper.

Bode plots of the graphene electrodes and of commercial Ag/AgCl electrodes within the 0.05 to 10 Hz frequency range are presented in Figure 6. As shown in this figure, the values of the impedance (R) decrease strongly with increasing frequency for both the graphene textile and the Ag/AgCl electrode; for the graphene–PET structure and the graphene paper electrode, however, the value of R shows negligible change with increasing frequency. Additionally, the impedance of the Ag/AgCl electrode increased obviously as the conductive gel dried over time (Ag/AgCl dry), which would directly affect the ECG signal detection sensitivity. At a frequency of 1 Hz (which is close to the normal human heart rate), the impedances of the graphene textile, the graphene–PET structure, the Ag/AgCl dry electrode, the Ag/AgCl wet electrode, and the graphene paper are 2.9 MΩ, 1.25 kΩ, 967 Ω, 388 Ω, and 19.8 Ω, respectively. The graphene paper has a much smaller intrinsic internal resistance than the other materials, thus demonstrating the excellent conductivity of few-layers graphene. The impedance of the graphene textile is higher than that of graphene paper, which may be a result of the number of graphene layers, the deposition and reduction amounts, and structural defects resulting from the grid interval of the polyester fiber. However, with control of the reaction process, we have reduced the impedance of the graphene textile electrode to dozens of kΩ.

**Figure 7:**
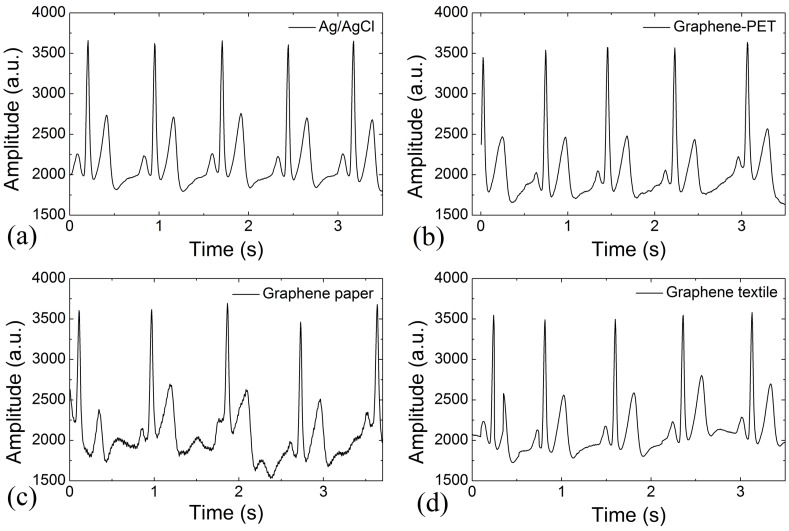
The ECG signals detected by the different types of electrodes. (**a**) commerical Ag/AgCl; (**b**) graphene–PET; (**c**) graphene paper; (**d**) graphene textile.

To estimate the ECG detection performances of the different kinds of graphene electrode, typical ECG signals are collected, as shown in Figure 7. Based on a comparison of the different kinds of electrodes, we discovered that the waveform of the ECG signal from the graphene–PET (Figure 7b), graphene paper (Figure 7c), and graphene textile (Figure 7d) electrodes were quite similar to that from the commercial Ag/AgCl electrode (Figure 7a), with no significant differences between their waveforms, amplitudes, and signal-to-noise ratios (SNRs). In Figure 7, the P, Q, R, S, and T waves all appeared clearly; the peak-to-peak amplitudes of these ECG signals were all approximately 1900 arbitrary units (a.u.), and their SNRs showed similar values of around 32 dB. These experiments demonstrate that while it has a larger intrinsic internal resistance, the synthesized graphene textile electrode is capable of detecting the ECG signals with high sensitivity.

**Figure 8:**
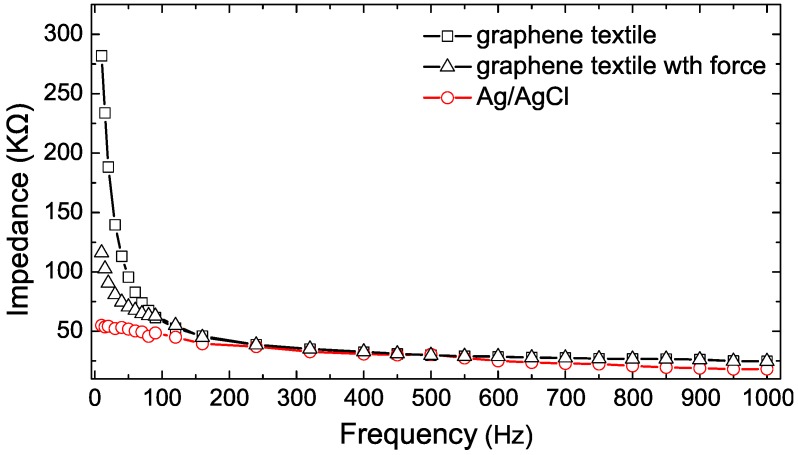
The skin–electrode contact impedance of Ag/AgCl and graphene textile electrodes with different contact force.

When compared with graphene–PET and graphene paper, the graphene textile electrode shows the best flexibility and assembly characteristics, and thus it was selected for further study in terms of its performance in daily ECG monitoring applications. Skin–electrode contact impedance directly influenced the reliability of collected signals, low contact impedance resulting in less noise and a higher quality ECG signal. Here, we measured the skin–electrode contact impedance of graphene textile with different contact force, and compared that to standard Ag/AgCl electrode. Figure 8 shows that the impedance values of the conventional Ag/AgCl electrode ranges from 55.1 kΩ (at 10 Hz) to 18.2 kΩ (at 1 kHz), similar to results reported in the literature [24], and the impedance of the graphene textile electrode varies from 281.8 kΩ (at 10 Hz) to 24.8 kΩ (at 1 kHz). The results show that the graphene textile electrode has higher skin–electrode contact impedance compared to the conventional Ag/AgCl electrode. It has also been observed that the contact impedance reduced obviously by increasing the skin–electrode contact force. When additional contact force was applied using compression bandages, the impedance of the graphene textile electrode varied from 116.2 kΩ (at 10 Hz) to 24.7 kΩ (at 1 kHz), close to that of the conventional Ag/AgCl electrode. The results imply that textile electrode should assist with consistent application of contact force to obtain high-quality signals.

**Figure 9:**
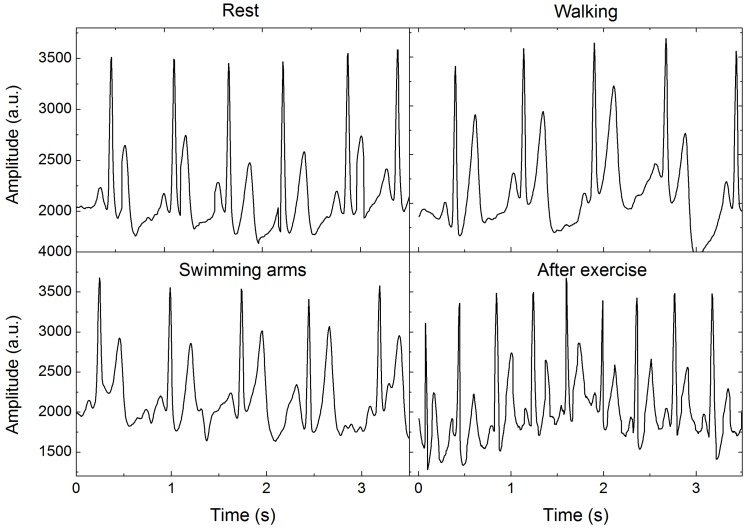
Comparison of ECGs recorded using graphene textile electrode while the subject was seated and resting, walking, swinging their arms, and exercising.

Four examples of motion artifacts are shown in Figure 9; these artifacts occurred as the subject rested, walked around at a velocity of around 1.3 m/s, swung their arms, and exercised (by running approximately 1 km). As shown in this figure, in the original ECG signals that were measured at rest, the P, Q, R, S, and T waves appeared clearly with high SNRs, and the heart rate was approximately 66 beats/min. Walking and swinging of the subject’s arms led to negligible drift and deformation of the ECG signals; both signals remained stable, and the P, R, and T peaks could be successfully discriminated. After exercise, the ECG signals can again be detected with high SNRs. While these signals have some baseline drift, each peak can be distinguished easily, no critical P-wave amplitude differences were observed, and the heart rate reached up to 150 beats/min, which was much higher than the rate before exercise.

Furthermore, we compared the performance of graphene textile electrode with commercial Ag/AgCl electrode at different motion states, the peak-to-peak amplitude (P–P), heart rate, SNRs, and standard deviation (SD) of collected ECG signals are summarized in Table 1. Baseline drift in ECG signal is the biggest hurdle in the visualization of correct waveform; in this paper, a measure of baseline drift is calculated by detecting and removing the Q, R, S, and T peaks and calculating the SD of the remaining data points. As displayed in this table, the two electrodes provide high signal quality and performance, and the calculated values agree with each other very well. The deviation is less than 5%, and the SNR of graphene textile is about 3% higher than that of the Ag/AgCl electrode. The SD statistics indicate that the noise and baseline drift levels are encountered during motion states for each electrode. The electrodes perform best at rest, having less baseline drift than motion state; walking and running exercise result in much more significant baseline drift than swimming arms. After running exercise, the value of SD for graphene textile and Ag/AgCl electrode were 267.8 and 276.3, respectively. Compared with Ag/AgCl electrode, less baseline drift was observed by graphene textile, possibly because perspiration can pass through the fabric avoiding the changes in skin impedance and skin–electrode contact impedance.

**Figure 10:**
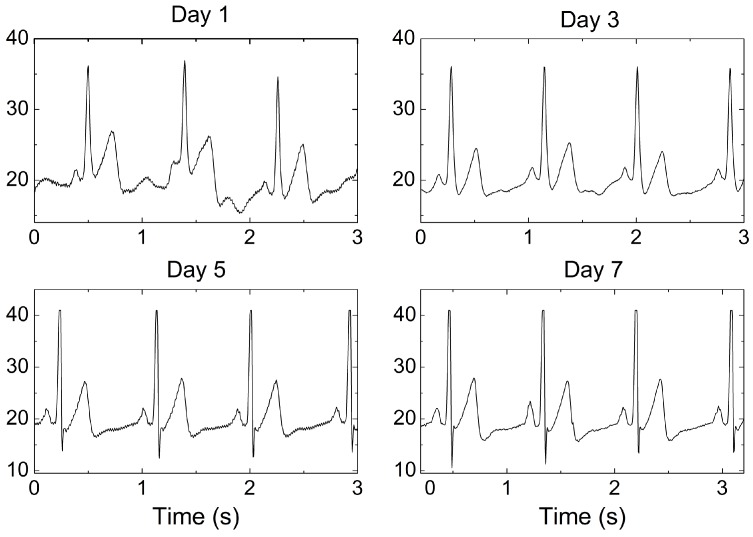
ECG signals measured after 1, 3, 5, and 7 days of wearing the electrode.

In order to assess the performance in long-term measurement, the ECG signal was collected over seven days of continuous wear of the graphene textile electrode. The waveforms of ECG signals in days 1, 3, 5, and 7 are displayed in Figure 10, and the full set of statistics on these recordings is given in Table 2. As shown in Figure 10, the the P, Q, R, S, and T waves all appeared clearly with high SNR; no degradation of the ECG signal amplitude and no baseline drift in the waveform were observed over time. The large deviation in P–P amplitude may have been a result of changes in the position and the contact resistance with the subject’s skin. As displayed in Table 2, the SNRs of ECG signals collected by graphene textile electrode with 7-day continuous wear reached up to about 29.2 dB, indicating the superior characteristics of this electrode for long-term monitoring applications. These results indicate that the flexible graphene textile electrode overcomes the problems that most wet electrodes have in terms of attenuation of their detection sensitivity, and are thus superior for long-term monitoring while providing comfortable wearability.

## 4. Discussion

Mobile health care systems have increased in popularity because of their noninvasive features and convenience for use in daily life. In this study, we aimed to develop wearable flexible electrodes for daily ECG monitoring, which would be very helpful in the detection of sudden onset heart disease, especially in elderly patients. Electrodes that are highly sensitive, comfortable, and offer biological compatibility and persistence are essential components for daily ECG monitoring systems. The results presented here have demonstrated that the proposed graphene-based electrode offers sensitivity comparable to that of commercial Ag/AgCl electrodes; this research also indicates opportunities to transfer graphene for use in other biological applications, such as electroencephalography (EEG) and electromyography (EMG) detection.

However, one challenge that remains for the use of graphene materials in daily ECG monitoring is their intrinsically high hydrophobicity, which might lead to a weak binding force to the substrate and high impedance between the graphene and electrolyte surfaces. In our early study of graphene electrodes on a soft elastic PDMS substrate, the graphene films were found to fall off easily, and they showed much higher resistance due to broken parts of the graphene structure. In this study, graphene was adsorbed on polyester fibers using a vacuum suction filter; the intrinsic internal resistance of the graphene textile reached several megaohms, indicating that the adsorption amount and the combination degree must be further improved to enhance the detection sensitivity and the frictional resistance. We will apply our efforts to the selection of a more suitable substrate, and will increase the binding force between the graphene films and the substrate by introducing molecules with grafting chains. With regard to toxicity, carbon is commonplace and has a stable structure; previous studies have demonstrated the biological compatibility of carbon group elements, including carbon nanotubes and graphene [30], and thus there is no doubt over the nontoxicity of graphene. While this paper is focused on ECG sensing, these graphene electrodes can also be applied to other electrophysiological sensing methods, such as EMG and EEG. Additionally, graphene has pressure sensitivity properties [31,32], and its impedance changes with temperature [33]; using a combination of these properties, the development of wearable integrated multidimensional physiological information detection sensors may be feasible.

## 5. Conclusions

In this paper, we have fabricated graphene-based electrodes and a corresponding wireless ECG collection system. The results demonstrated that ECG signal recording can be carried out using a flexible graphene electrode with high SNR. The graphene electrode provides effective electrical performance, high flexibility, satisfactory biocompatibility and wearability, and offers detection capabilities in various states of motion. The design presented here provides a potential electrode structure for long-term wearable monitoring, and offers convenience and comfort for home health care management of the elderly population, with collection and computer display of the results performed via wireless communication using the developed graphene electrode.

## Figures and Tables

**Table 1:** Peak-to-peak amplitude (P–P; a.u.), heart rate (bpm), signal-to-noise ratios (SNRs, dB), and standard deviation (SD) for graphene textile and Ag/AgCl electrode set at motion states.

Motion State	Graphene Textile				Ag–AgCl			
	P–P	Heart Rate	SNR	SD	P–P	Heart Rate	SNR	SD
Rest	1750	66	31.6	116.8	1790	66	31.0	104.2
Walking	1920	70	31.2	243.5	2010	71	30.1	233.6
Swimming arms	1900	68	29.5	166.3	1910	66	28.7	164.4
Exercised	1875	150	28.3	267.8	1834	147	28.5	276.3

**Table 2:** Peak-to-peak amplitude (P–P; a.u.) and signal-to-noise ratios (SNRs, dB) of detected ECG signals by graphene textile electrode within one week.

	Day 1	Day 2	Day 3	Day 4	Day 5	Day 6	Day 7
P–P	18.3	18.0	18.2	21.3	26.7	25.7	30.1
SNR	24.1	28.6	33.7	29.4	32.6	27.5	29.2

## Supplementary Materials

The following are available online at http://www.mdpi.com/1424-8220/16/11/1833/s1, Video S1: Realtime ECG collection and display in computer by wireless communication and the developed graphene electrode.

## Abbreviations

The following abbreviations are used in this manuscript:

ECG	electrocardiography
SEM	scanning electron microscope
SNR	signal-to-noise ratio
CNT	carbon nanotube
MWCNTs	multiwall carbon nanotube arrays
PDMS	polydimethylsiloxane
PET	polyethylene terephthalate
CVD	chemical vapor deposition
EMG	electromyography
EEG	electroencephalography
